# Meteorological drivers of respiratory syncytial virus infections in Singapore

**DOI:** 10.1038/s41598-020-76888-4

**Published:** 2020-11-24

**Authors:** Sheikh Taslim Ali, Clarence C. Tam, Benjamin J. Cowling, Kee Thai Yeo, Chee Fu Yung

**Affiliations:** 1 WHO Collaborating Centre for Infectious Disease Epidemiology and Control, School of Public Health, Li Ka Shing, Faculty of Medicine, The University of Hong Kong, Pok Fu Lam, Hong Kong Special Administrative Region China; 2 Saw Swee Hock School of Public Health, National University of Singapore and National University Health System, Tahir Foundation Building, 12 Science Drive 2, Singapore, 117549 Singapore; 3 London School of Hygiene and Tropical Medicine, London, UK; 4 Department of Neonatology, KK Women’s and Children’s Hospital, Singapore, Singapore; 5 Infectious Diseases Service, KK Women’s and Children’s Hospital, Singapore, Singapore; 6 Lee Kong Chian School of Medicine, Nanyang Technological University, Singapore, Singapore

**Keywords:** Risk factors, Infectious diseases, Respiratory tract diseases, Epidemiology

## Abstract

Meteorological drivers are known to affect transmissibility of respiratory viruses including respiratory syncytial virus (RSV), but there are few studies quantifying the role of these drivers. We used daily RSV hospitalization data to estimate the daily effective reproduction number (*R*_*t*_), a real-time measure of transmissibility, and examined its relationship with environmental drivers in Singapore from 2005 through 2015. We used multivariable regression models to quantify the proportion of the variance in *R*_*t*_ explained by each meteorological driver. After constructing a basic model for RSV seasonality, we found that by adding meteorological variables into this model we were able to explain a further 15% of the variance in RSV transmissibility. Lower and higher value of mean temperature, diurnal temperature range (DTR), precipitation and relative humidity were associated with increased RSV transmissibility, while higher value of maximum wind speed was correlated with decreased RSV transmissibility. We found that a number of meteorological drivers were associated with RSV transmissibility. While indoor conditions may differ from ambient outdoor conditions, our findings are indicative of a role of ambient temperature, humidity and wind speed in affecting RSV transmission that could be biological or could reflect indirect effects via the consequences on time spent indoors.

## Introduction

RSV is a significant public health problem that causes considerable morbidity and mortality each year, especially amongst infants and young children. An estimated of 33 million new episodes of respiratory syncytial virus (RSV)-associated acute lower respiratory infection occur in children < 5 years annually, resulting in 3 million hospitalisations and in 60,000 deaths^1^. There is increasing evidence that RSV is routinely underdiagnosed and can also cause significant disease burden and mortality in the elderly^2^. Understanding the metereological drivers of RSV transmissibility could allow early predictions of RSV epidemics to help healthcare facilities and hospitals prepare for surges in demand, or indicate potential opportunities for environmental control measures.

Besides intrinsic drivers of transmissibility, such as natural immunity, virus survival and antigenic variation^3^, seasonal meteorological drivers may affect RSV transmission, but their role is uncertain. One study conducted in multiple locations reported that a U-shaped association between humidity and RSV transmissibility could explain the occurrence of winter and summer epidemics^4^. In tropical settings, rain, humidity and temperature have been found to increase the incidence of RSV disease^5^. In other settings, RSV incidence has been found to be associated with low temperature^6–8^, high humidity^6,7^, and greater diurnal temperature range (DTR)^9^.

All these earlier studies on the climatic drivers of RSV transmission used absolute counts of RSV cases or hospital admissions as the dependent variable in their analysis. This is not an ideal measure of RSV transmission intensity, as absolute case counts do not necessarily represent the changes in the transmissibility or the proportion of susceptible individuals in the population. Instead, it is more informative to investigate associations between meteorological drivers and a more direct measure of RSV transmissibility in the community^10,11^. Here, we estimated the daily effective reproduction number (*R*_*t*_), a real-time measure of transmissibility for RSV infection, using hospitalization data from the tropical city of Singapore, which has near year-round circulation of RSV. We then examined the relationship between daily *R*_*t*_ values with potential meteorological drivers.

## Results

**Figure 1:**
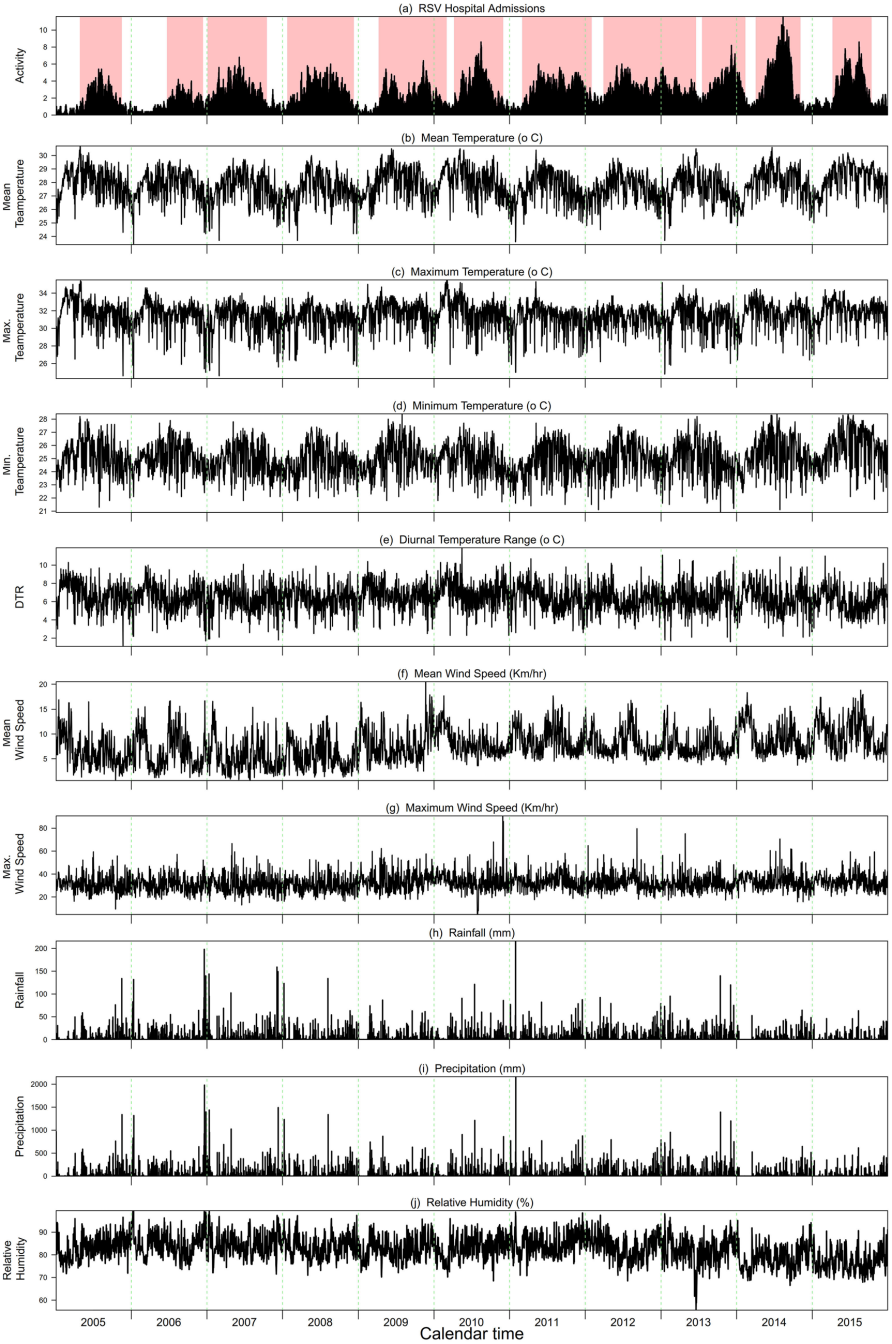
(**a**) Daily activity of RSV hospital admissions (black bars) along with the 11 predefined epidemics (light red bars); (**b**–**j**) the meteorological drivers in Singapore from 2005 through 2015.

The time series of RSV hospital admissions and meteorological drivers are shown in Fig. 1 for the period from January 2005 through December 2015. Descriptive measures for these time series are presented in Table S1. RSV circulated all year round with irregular timing of the onset seasonal epidemics. We identified 11 distinct RSV epidemics (with 14 observed peaks) with different lengths, which covered 73% of the total study period.

**Figure 2:**
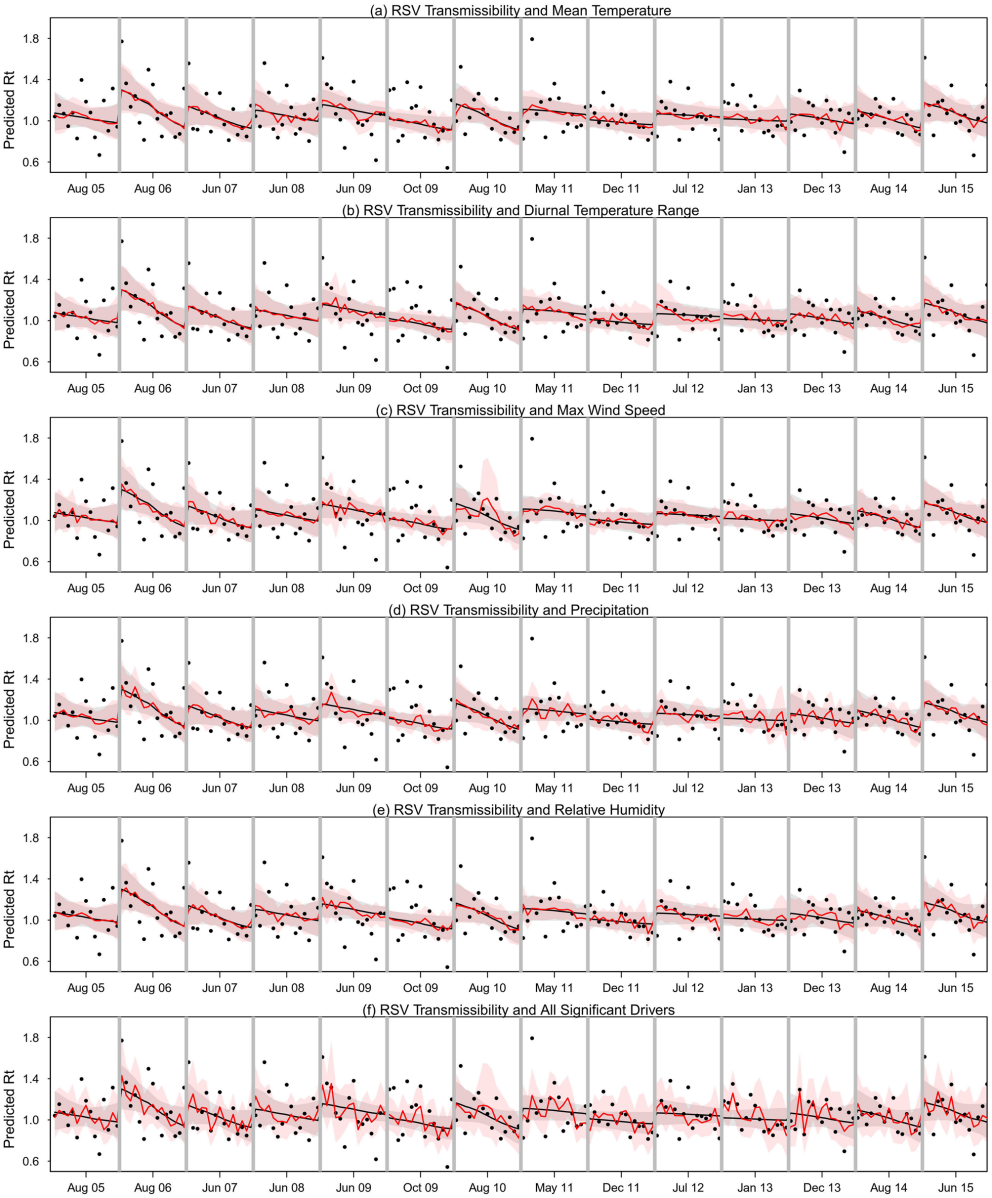
Effective reproduction numbers inferred from the RSV hospital admissions time series (black dots) and the predicted effective reproduction numbers from basic models with inclusion of depletion of susceptible and the inter-seasonal factors only (black lines) with 95% CI (light grey shaded area), and inclusion of meteorological drivers in addition to depletion of susceptible and the inter-seasonal factors (red lines) with 95% CI (light red shades). Total 11 epidemics (with 14 peaks) of RSV transmission in Singapore during 2005–2015. The difference between the black line and the red line illustrates the improvement in fitting due to inclusion of respective drivers. We used a maximum duration of 7 weeks to both side of peaks and a 5-days moving average window to smooth daily hospitalization data for distributed lag model (DLM) with lags of 0–14 days.

We quantified the *R*_*t*_ for RSV infections during the 11 epidemics (Fig. 2). The *R*_*t*_ values across all epidemics had a median of 1.03 (95% credible interval (CrI) 0.66, 1.52), with a maximum value of 2.32 (95% CrI 1.03, 4.39) at the start of one epidemic and a minimum value of 0.40 (95% CrI 0.21, 0.67). *R*_*t*_ generally declined over time during each epidemic (Fig. 2).

We explored the association between RSV transmissibility, as measured by *R*_*t*_ and each meteorological factor with lagged values of 0–14 days (Table S2, S3 and S4). The estimated AIC differences ($\Delta_{j}$) indicated that the exponential and power form of non-linear association better represented the effect of the meteorological drivers on RSV transmissibility (Table S4). RSV transmissibility was positively associated with diurnal temperature range, daily precipitation and relative humidity and had a J-shape association with daily mean temperature, maximum temperature, but was negatively associated with maximum wind speed (Figure S1). These significant drivers were included in further analysis.

In multivariable regression analysis, the model could explain 27% of the observed variation in estimated reproduction numbers (*R*_*t*_). A considerable fraction of the observed variance (11%) in *R*_*t*_ was explained by the basic model including depletion of susceptibles during the outbreak and inter-epidemic effects (Table 1, S5–S7). Inclusion of the meteorological drivers (with identified forms of association) in the multivariable regression model improved model fit (*R*^2^) marginally, explaining up to 15% of the variance in *R*_*t*_ (Fig. 2 and S2). Compared with the best lag models, the distributed lag models increased the variation explained by meteorological drivers by almost double (Table 1, S5–S7). Individually, mean temperature, diurnal temperature range, maximum wind speed, precipitation and relative humidity could explain a maximum of 3.40%, 2.29%, 4.85%, 4.13% and 3.84% of the variance in *R*_*t*_ respectively.

**Table 1:** Proportions of the variance of the effective reproduction number explained by the meteorological drivers, from models on pre-defined RSV epidemics with a maximum duration of 7 weeks to both side of peaks for RSV infections in Singapore from 2005 through 2015.

Drivers	Best lag model			Distributed lag model		
Models^b^	*R*^2^	$\% \,\Delta R^{2}$	*df*	*R*^2^	$\% \,\Delta R^{2}$	*df*
Depletion of susceptible (DS)	0.0207	–	162	0.0207	–	162
DS + inter Epidemic factor^a^	0.1139	9.32	152	0.1139	9.32	152
+ Mean Temperature	0.1303	1.64	150	0.1479	3.40	144
+ Diurnal temperature range	0.1206	1.35	150	0.1254	2.29	144
+ Maximum wind speed	0.1397	2.58	150	0.1625	4.85	144
+ Precipitation	0.1435	2.96	150	0.1552	4.13	144
+ Relative Humidity	0.1305	1.66	150	0.1523	3.84	144
+ All drivers^c^	0.1889	7.50	142	0.2673	15.34	116

We used the 5-daysmoving average window to smooth daily hospitalization data for best lag and distributed lag model with lags of 0–14 days.

^a^Basic model: factors affecting *R*_*t*_ include depletion of susceptibles, inter-epidemic factors.

^b^Improved models include the basic model for *R*_*t*_ plus the respective drivers.

^c^Improved model includes the drivers: mean temperature, diurnal temperature range, maximum Wind Speed, Precipitation and Relative Humidity (statistically significant and free from multicollinearity). $\% \,\Delta R^{2}$ measured the change in the explained variance (of total variance) from the model in comparison to the basic model.

**Figure 3:**
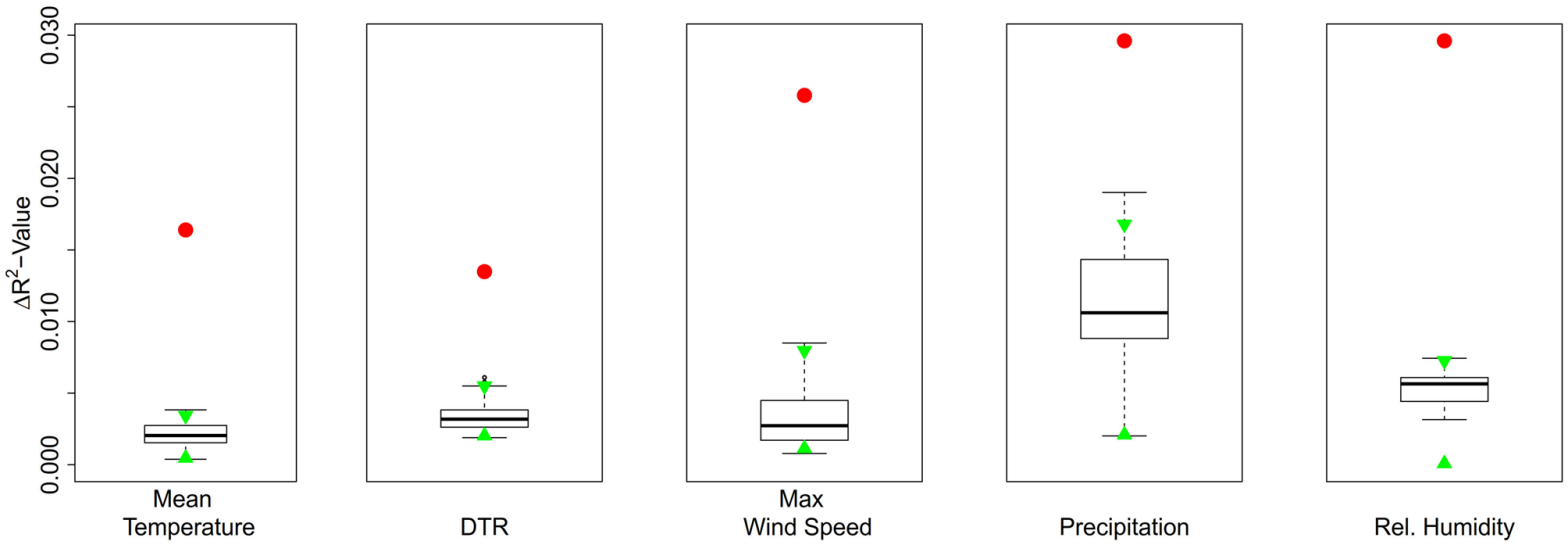
Permutation test representing the proportions of the variance ($\Delta R^{2}$) in the effective reproduction number explained by the true and 1000 null/dummy time series of respective significant drivers. The boxplot represents the $\Delta R^{2}$ from 1000 null/dummy time series generated by permutation on the years, and the stand-alone red points are indicating $\Delta R^{2}$ by true time series of the drivers. The green triangles show the 95% CI of $\Delta R^{2}$ evaluated by these 1000 null/dummy time series.

In a sensitivity analysis we varied the degree of smoothing applied to the daily RSV hospital admission data (0, 3, 5 and 7-days moving averages), and found no substantive change in the results (Table S5). Additionally, a sensitivity analysis including data from 5, 7 or 9 weeks either side of the epidemic peak led to similar findings (Table S6–S7). The permutation analysis on the 1000 null/dummy time series of these meteorological drivers in Singapore was able to explain substantially less variance in transmissibility compared to the observed true time series (Fig. 3).

## Discussion

We investigated the association between meteorological factors and RSV transmissibility in Singapore. Diurnal temperature range, daily precipitation and relative humidity are positively associated with RSV transmissibility, while maximum wind speed was negatively associated with transmissibility. Daily mean temperature and maximum temperature had a J-shape form of association with RSV transmissibility. Although a considerable proportion of the variance was attributable to intrinsic factors in the basic model for RSV transmissibility, the meteorological drivers were found to explain a further 15% of the total variance in RSV transmissibility in Singapore.

The positive relationship between DTR and RSV transmissibility was consistent with a previous study from Japan^9^ and could be associated with temperature-related changes in immunity^12,13^. In agreement with other studies^14,15^, we found that RSV transmissibility was negatively associated with maximum wind speed. Higher precipitation and relative humidity were associated with increased RSV transmissibility, potentially because of biological associations with virus survivability^5,6^. Another possible interpretation for our observations is that ambient weather is associated with the amount of time spent indoors when it is wet or windy outside. Time spent indoors in closed or confined environments could theoretically facilitate the transmission of respiratory viral infections such as RSV. In tropical settings, air-conditioning facilities in childcare centres and hospitals or clinics are not uncommon especially in cities. Public transport infrastructures especially underground trains are also air-conditioned. Since temperature and humidity could potentially be controlled by air-conditioners, it may be possible to mitigate the risk of RSV transmission in such settings. A more detailed understanding of this relationship will be important to inform such measures.

A potential limitation of our study is that RSV hospital admission data may not be an accurate indicator of RSV transmission in the community. However, although hospitalization data underestimate the overall burden of RSV, we would expect that the seasonal pattern of hospitalizations reflect the general temporal trend in RSV transmission in the community. In our study, we were unable to model the effect of air pollutants, as these data were not available to us. Air pollutants have been found in other studies to be associated with emergency department visits for respiratory conditions which include both infective and non-infective causes^8,16,17^. Finally, spatial averaging of outcome and weather data may have limited our ability to detect associations, as weather variables may not reflect conditions at the location where each case was located. However, Singapore is a small island with limited spatial variation in weather patterns or might change equally across the country over time.

Our analysis had a number of notable strengths. Firstly, all paediatric respiratory admissions at KKH are routinely tested for RSV, minimizing the risk of diagnostic bias that could result if, for example, clinicians were more likely to request RSV testing during times of high-suspected RSV transmission. Secondly, by estimating the instantaneous reproduction number, *R*_*t*_ we were able to model the effects of temperature on a more direct measure of transmissibility and allow for changes in transmissibility over time. We accounted for a range of functional forms and lagged effects to examine the association between *R*_*t*_ and meteorological drivers, and we performed several sensitivity analyses to assess the impact of data smoothing and epidemic timing on our results. We additionally performed extensive permutation tests that indicated that the observed association were unlikely to be due to chance (Fig. 3 and Appendix, section 3).

In conclusion, by using RSV transmissibility instead of reported cases, we identified a number of meteorological drivers of RSV infection. These included diurnal temperature range, daily precipitation, relative humidity, maximum wind speed, daily mean temperature and maximum temperature. Together they account for approximately 15% of RSV variance over time. Our findings will be an aid to develop predictive models to address the complex mechanism of RSV transmission in the tropics and subtropics.

## Materials and methods

### Data on RSV hospitalizations

We retrieved the daily number of RSV-related hospitalizations among children aged up to 30 months admitted to KK Women’s and Children’s Hospital (KKH) in Singapore between 2005 and 2015. KKH is the largest specialist women’s and children’s public hospital in Singapore. Data on positive identifications of RSV were extracted from laboratory diagnostics records at KKH. Paediatric respiratory admissions are routinely tested for range of respiratory pathogens, including direct fluorescence antibody (DFA) for influenza A, influenza B, RSV, adenovirus, parainfluenza viruses 1, 2 and 3, and human metapneumovirus (D3 Double Duet DFA Respiratory Virus Screening & ID Kit; Diagnostic Hybrids, Athens, OH, USA).

We used the daily time series of RSV hospitalisations as a measure of RSV activity in the community. We used a 3, 5,and 7-days moving average to smooth the time-series of hospital admissions to avoid zero values during the peak seasons.

We then defined RSV epidemic onsets as periods of seven or more consecutive weeks during which an epidemic threshold was exceeded for RSV hospitalizations. The epidemic threshold was determined as the 50th percentile of all the non-zero daily hospitalizations over the study period (Fig. 1a)^11^.

### Meteorological data

The daily mean air temperature, minimum air temperature, maximum air temperature, mean rainfall, mean wind speed, maximum wind speed, precipitation and relative humidity were obtained from the National Environment Agency, Singapore (https://www.weather.gov.sg/climate-historical-daily/). We assumed minimal spatial variability for these meteorological variables in the small island country, Singapore, and hence for simplicity, we considered the data from Changi station only for our analysis (Fig. 1b–j). We derived the daily diurnal temperature range (DTR) from the daily minimum and maximum air temperature, and computed daily absolute humidity from daily relative humidity and mean temperature.

### Estimation of the daily effective reproductive number, *R*_*t*_

Transmissibility can be measured by the effective (or instantaneous) reproduction number (*R*_*t*_), defined as the average number of secondary infections caused by a typical single infectious person at time *t*. We estimated *R*_*t*_ from daily numbers of hospital admissions of RSV disease using a simple branching process model^18^. We assumed the serial interval for RSV followed a Gamma distribution with a mean of 7 days and a standard deviation of 3.5 days^19–21^ (Appendix 1).

### Exploratory data analysis

We first assessed the best-fitting functional forms for the association between *R*_*t*_ and each meteorological driver by fitting linear, exponential and power forms for each weather variable at lags of 0 to 14 days. The best-fitting functional form was selected based on the Akaike Information Criterion (AIC).

### Multivariable regression analysis

We used a multivariable log-linear regression model for our analysis as described in Appendix 2. Our basic model incorporated depletion of susceptibles over time and inter-epidemic effects. We then fitted improved models including the significant meteorological drivers. We quantified the impact of individual drivers by comparing R-square (*R*^2^) values for the basic and improved models. We evaluated *R*^2^-values by using the best lag (i.e. the lag for which the model has the largest *R*^2^-value) and a distributed lag model (DLM, through the dlnm package in R), where the latter summarizes the overall effect distributed over multiple days instead of just reporting the results with the single best lag. We considered data from up to 7 weeks either side of the peak in the multivariable regression models to avoid the effects of the low and irregular reporting during the very beginning and end of each epidemic, and conducted sensitivity analyses with a maximum of 5 and 9 weeks either side of the peak. To assess whether these quantified associations were due to chance, we performed permutation analysis on multivariable regression models with 1000 dummy or null scenarios and compared the results with one real time series (details in Appendix 3)*.*

### Ethics statement

Ethical approval and waiver of informed consent for this secondary analysis of anonymized data was obtained from SingHealth’s Central Institutional Review Board (application number: 201702-00121). This study is reported in accordance with STROBE guidelines for observational studies.

## Supplementary information

Supplementary Information.
