# Supplementary material for: Meteorological drivers of respiratory syncytial virus infections in Singapore
Source: Sci Rep. 2020 Nov 24;10:20469. doi: 10.1038/s41598-020-76888-4 (PMC7686497; doi:10.1038/s41598-020-76888-4)
Supplement: Supplementary file 1 — Supplementary Information. [file 41598_2020_76888_MOESM1_ESM.docx]

**Supplementary Information – Meteorological drivers of respiratory syncytial virus infections in Singapore**

**Authors:**

Sheikh Taslim Ali^1^, Clarence C Tam^2,3^, Benjamin J Cowling^1^, Kee Thai Yeo^4,5^, Chee Fu Yung^4,5,6^

**Affiliations:**

^1^ WHO Collaborating Centre for Infectious Disease Epidemiology and Control, School of Public Health, Li Ka Shing Faculty of Medicine, The University of Hong Kong, Hong Kong Special Administrative Region, China

^2^ Saw Swee Hock School of Public Health, National University of Singapore and National University Health System, Singapore

^3^ London School of Hygiene & Tropical Medicine, London, United Kingdom

^4^ Department of Neonatology, KK Women’s and Children’s Hospital, Singapore

^5^ Infectious Diseases Service, KK Women’s and Children’s Hospital, Singapore

^6^ Lee Kong Chian School of Medicine, Nanyang Technological University, Singapore

**Corresponding Author:**

Clarence C Tam

Saw Swee Hock School of Public Health, Tahir Foundation Building, 12 Science Drive 2, Singapore 117549, Singapore. Email: clarence.tam@nus.edu.sg

Running head: Meteorological drivers and RSV

**CONTENTS**

1. **Estimation of Transmissibility**
2. **Construction of Multivariable Regression Models**
3. **Permutation analysis for causal evidence on the predicted association between transmissibility and drivers.**
4. **Supplementary tables**
   1. *Table S1*
   2. *Table S2*
   3. *Table S3*
   4. *Table S4*
   5. *Table S5*
   6. *Table S6*
   7. *Table S7*
5. **Supplementary figures**
   1. *Figure S1.*
   2. *Figure S2.*
6. **Estimation of Transmissibility**

We estimated the daily instantaneous reproduction numbers $R_{t}$ following the Bayesian framework applied on the branching process models, proposed by Cori et. al. [10], which is an extension of Fraser’s method [21]. Fraser proposed the time varying estimate of $R_{t}$ from the deterministic renewal equation of an epidemic as

$$R_{t}=\frac{I_{t}}{\sum_{k=0}^{m} w_{k}I_{t-k}} (1.1)$$

where $I_{t}$ is the number of incident symptomatic cases (here, RSV hospital admissions) between time $t$and time $t+1$ and $w_{k}$ the serial interval distribution, such that $\sum_{k=0}^{m} w_{k}=1$. The serial interval distribution is assumed to be constant during the epidemic. A Bayesian framework was developed to generalize this inferential approach and to account for the inherent stochasticity of the transmission process, assuming the expected incidence at time $t$ ($R_{t}\sum_{k=0}^{m} w_{k}I_{t-k}$), is a Poisson-distributed count. Consider, the transmissibility is constant over the time window [$t-\tau, t$], and denoted by $R_{[t-\tau,t]}$; then the likelihood of $I_{t-\tau}, \ldots\ldots\ldots,I_{t}$ given $R_{[t-\tau,t]}$ and $I_{0}, \ldots\ldots\ldots,I_{t-\tau-1}$ is as

$${P(I}_{t-\tau}, \ldots\ldots\ldots,I_{t}\left| I_{0}, \ldots\ldots\ldots,I_{t-\tau-1}, w,R_{\left[ t-\tau,t \right]}) \right.=\prod_{s=t-\tau}^{t} \frac{{{e^{-R_{\left[ t-\tau,t \right]}\Lambda_{s}} (R}_{\left[ t-\tau,t \right]}\Lambda_{s})}^{I_{s}}}{I_{s}!} (1.2)$$

where, $\Lambda_{s}=\sum_{s=0}^{m} w_{s}I_{t-s}$. Assuming, Gamma $(a,b)$ as a conjugate prior distribution for $R_{\left[ t-\tau,t \right]}$, the posterior joint distribution of $R_{\left[ t-\tau,t \right]}$ can be derived as proportional to

$${R_{\left[ t-\tau,t \right]}}^{a+\sum_{s=t-\tau}^{t} I_{s}-1} e^{-R_{\left[ t-\tau,t \right]}(\sum_{s=t-\tau}^{t} \Lambda_{s}+\frac{1}{b})} \prod_{s=t-\tau}^{t} \frac{{\Lambda_{s}}^{I_{s}}}{I_{s}!} (1.3)$$

The equation 1.3 indicates the posterior distribution of $R_{\left[ t-\tau,t \right]}$is a Gamma distribution with parameters$(a+\sum_{s=t-\tau}^{t} I_{s},{(\sum_{s=t-\tau}^{t} \Lambda_{s}+\frac{1}{b})}^{-1})$.

*Choice of Serial Interval (SI) distributions*: In this study, we assumed a Gamma distribution for the serial interval (SI) with the mean 7 days (SD=3.5 days) for RSV transmission [11-13].

1. **Construction of Multivariable Regression Models**

Transmissibility of respiratory viruses is generally influenced by the depletion of susceptibles and inter-epidemic effects as intrinsic drivers, along with the potential effects of the extrinsic drivers (e.g. meteorological drivers). Therefore, we used general multivariable nonlinear regression models to investigate further the underlying association between the transmissibility of RSV and different climatic drivers in Singapore.

We tested different regression forms to investigate the underlying association between the transmissibility of RSV and different plausible driving forces. We identified that the exponential form (i.e. $R_{t}=e^{e^{\varphi(f_{k})}}$, where, $\varphi\left( f_{k} \right)=\beta_{0}+\beta_{1}f_{k}+\beta_{2}{f_{k}}^{2}$, and power from ($R_{t}=\beta_{0}^{*}{f_{k}}^{\beta_{1}^{*}}$, where $\beta_{i}$’s and $\beta_{i}^{*}$’s are respective coefficients, $f_{k}$ are the $k$-th drivers) of association are better representation for across all the drivers with RSV transmissibility in Singapore (Table S4).

Following the epidemic model theory and the from above results, we construct a general multivariable nonlinear regression model described by te Beest et al [8]. Consider the$S_{ij}$ is the susceptibles (fraction) at the start of $i$th day/weeks of $j$th epidemic and $R_{0}$ is the basic reproduction number. Therefore, the effective reproduction number $R_{ij}$ can be written as,

$$R_{ij}=R_{0}S_{ij}\prod_{k} {f_{ijk}}^{\gamma_{k}}\prod_{l} e^{\varphi(g_{ijl})} (2.1)$$

Where, $R_{ij}$ is the time-varying instantaneous reproduction number on day $i$ of epidemic$j$. The effect of the driving factors ($f_{ijk}$ or$g_{ijl}$) during day $i$ for the epidemic$j$, is determined by the respective coefficients.

Let, $h_{ij}$ is the observed cumulative incidence up to $(i-1)$th day of $j$th epidemic. i.e. $h_{ij}=\sum_{x}^{i-1} I_{xj}$, where $I_{xj}$ is the incidence in day $x$ of epidemic $j$.

Hence, $S_{ij}=S_{0j}-{h_{ij}= S}_{0j}\left( 1+z_{j} h_{ij} \right),$where $z_{j}={-c_{i}/S}_{0j}$ with constant$c_{i}$ (here$,c_{i}=1$). $S_{0j}$ is initial susceptible proportion for epidemic $j$.

Now, from Taylor series, we have,$S_{ij}\approx S_{0j}e^{z_{j} h_{ij}}$ and finally we get,

$$R_{ij}=R_{0}S_{0j}e^{z_{j} h_{ij}} \prod_{k} {f_{ijk}}^{\gamma_{k}}\prod_{l} e^{\varphi(g_{ijl})} (2.2)$$

Taking logarithm we have,

$$ln\left( R_{ij} \right)=\ln\left( R_{0}S_{0j} \right)+z_{j} h_{ij}+\sum_{k} \gamma_{k} ln(f_{ijk})+\sum_{l} \varphi(g_{ijl}){+ \epsilon}_{ij}$$

In our analysis, we treated the parameters $\ln\left( R_{0}S_{0j} \right)$ and $z_{j}$ as the nuisance parameters, which generally indicated the inter-seasonal factors, and might not be of immediate interest. $\epsilon_{ij}\sim N(0,\sigma^{2})$ is the error term.

Therefore, we reasonably replaced with respective terms and constructed the following regression equation for our analysis,

$$ln\left( R_{ij} \right)=\alpha_{j}s_{j}+\eta_{j}h_{ij}+ \sum_{k} \gamma_{k} ln(f_{ijk}) +\sum_{l} \varphi(g_{ijl})+\epsilon_{ij} (2.3)$$

Where, $s_{j}$ indicates the epidemic$j$, a categorical variable with the regression coefficient $\alpha_{j}$ for the epidemic and $\eta_{j}$ is the respective regression coefficients indicating how quickly susceptibles deplete in the population.

We finally define the basic model is based on intrinsic factors only. i.e. $ln\left( R_{ij} \right)=\alpha_{j}s_{j}+\eta_{j}h_{ij}+\epsilon_{ij}$. Therefore, these $\Delta R^{2}$ measures (comparing the R-square values of these models) indicate the variance in transmissibility explained by respective drivers.

1. **Permutation Analysis for causal evidence on the predicted association between transmissibility and drivers.**

As the regression doesn’t confirm the causal evidence of predicted association between the response and predictor variables. In time series analysis, addressing causality is not always straight forward. In literature, to address this issue, researcher often uses a permutation analysis, which is much simpler and easy to interpret. In this analysis, the outcome measure (e.g. goodness of fit, R-squared etc.) is evaluated by allowing a wide range of noise in the predictor variables (null/dummy time series of predictors), and finally, comparing these outcomes with the outcomes derived through true time series. If the true time series of predictors provide better results than that of by null/dummy time series, indicates the evidence of causality.

***Permutation analysis:*** To test the causality on association between transmissibility and each meteorological drivers in Singapore, we used the simple permutation analysis.

1. Firstly, we have derived n=1000 permutation of true time series of each drivers by years.
2. Then we run n=1000 multivariable regressions (above models) on these 1000 null/dummy time series as predictor for respective drivers. Calculated R-squared ($R^{2}$) value for each 1000 sets of tome series.
3. Finally, evaluated 1000 $\Delta R^{2}$ by considering the differences of $R^{2}$. Where, the basic model is based on intrinsic factors only. i.e. $ln\left( R_{ij} \right)=\alpha_{j}s_{j}+\eta_{j}h_{ij}+\epsilon_{ij}$. Therefore, these $\Delta R^{2}$ measures indicate the variance in transmissibility explained by respective drivers.
4. Presentation: Generated the figures of the density of 1000 null/dummy $\Delta R^{2}$ along with one true $\Delta R^{2}$, which is derived using the true time series of significant meteorological drivers in Siongapore (see Figure. 3).

We find the true time series of these drivers explained more variance in transmissibility compare to that of by null/dummy time series. The red solid point in boxplot, indicates the value of $\Delta R^{2}$ (derived from true time series) which is higher than the median value of $\Delta R^{2}$ derived from null/dummy time series of the drivers (Figure 3).

**4. Supplementary tables**

**Table S1:** Summary of the variables derived from the daily time series on respiratory syncytial virus (RSV) related hospital admissions and meteorological drivers in Singapore during 2005-2015.

| Variables | | Mean | SD | Minimum | Percentile | | | Maximum |
| --- | --- | --- | --- | --- | --- | --- | --- | --- |
|  |  |  |  |  | **25th** | **Median** | **75th** |  |
| Age of RSV Hospital  Admissions | **Male**  **(n= 5804)** | 10.19 | 7.91 | 0.18 | 3.60 | 8.00 | 15.19 | 30.00 |
|  | **Female**  **(n=4101 )** | 10.78 | 8.28 | 0.15 | 3.34 | 8.79 | 16.98 | 30.00 |
|  | **All**  **(n= 9905)** | 10.44 | 8.07 | 0.15 | 3.51 | 8.27 | 16.02 | 30.00 |
| Meteorological Drivers | **Mean**  **Temperature** | 27.79 | 1.12 | 23.40 | 27.00 | 27.80 | 28.70 | 30.70 |
|  | **Max**  **Temperature** | 31.48 | 1.53 | 24.30 | 30.70 | 31.70 | 32.50 | 35.50 |
|  | **Min**  **Temperature** | 25.03 | 1.25 | 20.90 | 24.10 | 25.00 | 25.90 | 28.40 |
|  | **Mean Wind Speed** | 7.60 | 3.24 | 0.60 | 5.40 | 7.10 | 9.70 | 20.50 |
|  | **Max. Wind Speed** | 32.23 | 7.20 | 4.70 | 27.40 | 31.70 | 36.00 | 90.70 |
|  | **Rainfall** | 5.98 | 14.53 | 0.00 | 0.00 | 0.00 | 4.50 | 216.20 |
|  | **Precipitation** | 59.15 | 142.90 | 0.00 | 0.00 | 0.00 | 44.50 | 2162.00 |
|  | **Relative Humidity** | 82.39 | 5.61 | 55.60 | 78.60 | 82.40 | 86.20 | 99.50 |

**Table S2:** Summary of correlation test with 0-14 lag (in days): Measure of linear association between transmissibility (i.e.$\boldsymbol{R}_{\boldsymbol{t}}$) and meteorological drivers in Singapore during 2005-2015.

| Drivers | r [Range] | Significant Lags (Days) |
| --- | --- | --- |
| Mean Temperature | 0.03 [0.04, 0.06] | 1-9 |
| Max Temperature | 0.06 [0.05, 0.10] | 0-13 |
| Min Temperature | 0.01 [0.03, 0.04] | 6 |
| Diurnal temperature range | 0.06 [0.05, 0.11] | 0-13 |
| Mean Wind Speed | - 0.04 [-0.06, - 0.03] | 0-10 |
| Max. Wind Speed | - 0.05 [-0.07, - 0.03] | 1-9 |
| Rainfall | 0.05 [0.01, 0.07] | * |
| Precipitation | 0.05 [0.02, 0.08] | 4-5 |
| Relative Humidity | 0.02 [0.01, 0.06] | 10-14 |
| Absolute Humidity | 0.08 [0.07, 0.11] | 0-14 |

Correlation coefficients (r) for 0-lag and the range of r with lag of 1-14 days in brackets. * indicates non-significant measures

**Table S3:** Summary of univariate regression with 0-14 lag (in days): Measure of non-linear association between transmissibility (i.e.$\boldsymbol{R}_{\boldsymbol{t}}$) and meteorological drivers in Singapore during 2005-2015.

| Drivers | R^2^ [Range] | Significant Lags (Days) |
| --- | --- | --- |
| Mean Temperature | 0.0021 [0.0000, 0.0035] | 0-3 |
| Max Temperature | 0.0098 [0.0047, 0.0120] | 0-11 |
| Min Temperature | 0.0003 [0.0000, 0.0015] | * |
| Diurnal temperature range | 0.0035 [0.0027, 0.0070] | 0-4 |
| Mean Wind Speed | 0.0001 [0.0001, 0.0005] | * |
| Max. Wind Speed | 0.0049 [0.0045, 0.0104] | 1-12 |
| Rainfall | 0.0002 [0.0003, 0.0033] | * |
| Precipitation | 0.0001 [0.0001, 0.0005] | 4-5 |
| Relative Humidity | 0.0020 [0.0023, 0.0059] | 0-11 |
| Absolute Humidity | 0.0067 [0.0067, 0.0127] | * |

R-Square (R^2^) for 0-lag and the range of r with lag of 1-14 days in brackets. * indicates non-significant measures

**Table S4:** $\Delta\boldsymbol{AIC}$ values for three different forms of univariate regression models to identify the best model representing the association between effective reproduction number ($\boldsymbol{R}_{\boldsymbol{t}}$) for meteorological drivers of RSV in Singapore.

| Drivers | $\boldsymbol{\Delta}\boldsymbol{AIC}$ for associations of RSV and drivers | | |
| --- | --- | --- | --- |
|  | Linear | Exponential | Power |
| Mean Temperature | 517.14 | 0.00 | 1.02 |
| Max Temperature | 530.93 | 0.00 | 18.64 |
| Min Temperature | 517.86 | 0.00 | 0.17 |
| Diurnal temperature range | 512.25 | 0.00 | 1.27 |
| Mean Wind Speed | 518.52 | 2.73 | 0.00 |
| Max. Wind Speed | 526.14 | 0.00 | 10.97 |
| Rainfall | 517.71 | 1.78 | 0.00 |
| Precipitation | 517.19 | 0.75 | 0.00 |
| Relative Humidity | 517.72 | 0.00 | 0.36 |
| Absolute Humidity | 517.19 | 1.71 | 0.00 |

Note:$\Delta{AIC}_{i}={AIC}_{i}-{AIC}_{min}, {AIC}_{min}=min({AIC}_{Linear}, {AIC}_{Exponential}, {AIC}_{Power})$,$i=Linear, Exponential, Power$.

**Table S5.** Percentage of the variance of the effective reproduction number explained by the meteorological drivers, from models on pre-defined RSV epidemics with a maximum duration of 7 weeks to both side of peaks for RSV infections in Singapore from 2005 through 2015. The results based on 0, 3, 5 and 7 days moving average window to smooth daily hospitalization data for best lag and distributed lag model with lags of 0-14 days.

| Drivers  (Models *) | Best Lag Model | | | | | Distributed Lag Model | | | | |
| --- | --- | --- | --- | --- | --- | --- | --- | --- | --- | --- |
|  | 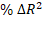 | | | | 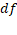 | 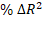 | | | | 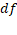 |
|  | **0** | **3** | **5** | **7** |  | **0** | **3** | **5** | **7** |  |
| Depletion of Susceptible (DS) | 1.93 | 1.99 | 2.07 | 2.23 | 162 | 1.93 | 1.99 | 2.07 | 2.23 | 162 |
| DS + Inter Epidemic factor† | 8.06 | 8.56 | 9.32 | 10.47 | 152 | 8.06 | 8.56 | 9.32 | 10.47 | 152 |
| + Mean Temperature | 1.56 | 1.62 | 1.64 | 1.6 | 150 | 3.35 | 3.39 | 3.4 | 3.39 | 144 |
| + Diurnal temperature range | 1.18 | 1.19 | 1.35 | 1.51 | 150 | 2.32 | 2.26 | 2.29 | 2.37 | 144 |
| + Maximum Wind Speed | 2.7 | 2.66 | 2.58 | 2.34 | 150 | 5.47 | 5.12 | 4.85 | 4.44 | 144 |
| + Precipitation | 3.48 | 3.25 | 2.96 | 2.64 | 150 | 4.98 | 4.55 | 4.13 | 3.68 | 144 |
| + Relative Humidity | 1.96 | 1.78 | 1.66 | 1.60 | 150 | 4.06 | 3.87 | 3.48 | 3.98 | 144 |
| + All drivers# | 7.83 | 7.01 | 7.50 | 7.25 | 142 | 16.76 | 16.06 | 15.34 | 14.45 | 116 |

† Basic Model: factors affecting $R_{t}$ include depletion of susceptibles, inter-epidemic factors. * Improved models include the basic model for $R_{t}$plus the respective drivers. # Improved model includes the drivers: mean temperature, diurnal temperature range, maximum Wind Speed, Precipitation and Relative Humidity (statistically significant and make sure to free from multicollinearity). ${\% \Delta R}^{2}$ measured the change in the explained variance (of total variance) from the model in comparison to the basic model. ${{\%}_{imp}\Delta R}^{2}$ measured the change in the explained variance (of variance explained by improved model) from the model in comparison to the basic model. i.e. $\% {\Delta R}^{2}=(R_{improved models}^{2}-R_{basic model}^{2})\times100$

**Table S6.** Proportions of the variance of the effective reproduction number explained by the meteorological drivers, from models on pre-defined RSV epidemics with a maximum duration of 9 weeks to both side of peaks for RSV infections in Singapore from 2005 through 2015. We used the 5 days moving average window to smooth daily hospitalization data for best lag and distributed lag model with lags of 0-14 days.

| Drivers | Best Lag Model | | | | Distributed Lag Model | | |
| --- | --- | --- | --- | --- | --- | --- | --- |
| (Models *) | 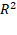 | 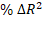 | 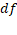 | 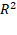 | | 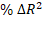 | 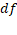 |
| Depletion of Susceptible (DS) | 0.0302 | - | 209 | 0.0302 | | - | 209 |
| DS+Inter Epidemic factor† | 0.1933 | 16.31 | 199 | 0.1933 | | 16.31 | 199 |
| + Mean Temperature | 0.2223 | 2.90 | 197 | 0.2428 | | 4.95 | 191 |
| + Diurnal temperature range | 0.1965 | 0.93 | 197 | 0.2033 | | 2.81 | 191 |
| + Max. Wind Speed | 0.2003 | 0.69 | 197 | 0.2099 | | 1.66 | 191 |
| + Precipitation | 0.2274 | 3.41 | 197 | 0.2654 | | 7.21 | 191 |
| + Relative Humidity | 0.2195 | 2.62 | 197 | 0.2393 | | 4.59 | 191 |
| + All drivers# | 0.2733 | 8.00 | 189 | 0.3572 | | 16.39 | 163 |

† Basic Model: factors affecting $R_{t}$ include depletion of susceptibles, inter-epidemic factors. * Improved models include the basic model for $R_{t}$plus the respective drivers. # Improved model includes the drivers: mean temperature, diurnal temperature range, maximum Wind Speed, Precipitation and Relative Humidity (statistically significant and make sure to free from multicollinearity). ${\% \Delta R}^{2}$ measured the change in the explained variance (of total variance) from the model in comparison to the basic model. ${{\%}_{imp}\Delta R}^{2}$ measured the change in the explained variance (of variance explained by improved model) from the model in comparison to the basic model. i.e. $\% {\Delta R}^{2}=(R_{improved models}^{2}-R_{basic model}^{2})\times100$

**Table S7.** Proportions of the variance of the effective reproduction number explained by the meteorological drivers, from models on pre-defined RSV epidemics with a maximum duration of 5 weeks to both side of peaks for RSV infections in Singapore from 2005 through 2015. We used the 5 days moving average window to smooth daily hospitalization data for best lag and distributed lag model with lags of 0-14 days.

| Drivers  (Models *) | Best Lag Model | | | Distributed Lag Model | | |
| --- | --- | --- | --- | --- | --- | --- |
|  | 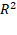 | 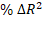 | 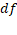 | 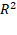 | 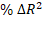 | 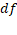 |
| Depletion of Susceptible (DS) | 0.0620 | - | 114 | 0.062 | - | 114 |
| DS + Inter Epidemic factor† | 0.1868 | 12.48 | 104 | 0.1868 | 12.48 | 104 |
| + Mean Temperature | 0.2030 | 1.62 | 102 | 0.2191 | 3.23 | 96 |
| + Diurnal temperature range | 0.2074 | 1.6 | 102 | 0.2151 | 3.02 | 96 |
| + Max. Wind Speed | 0.2262 | 3.94 | 102 | 0.2661 | 7.92 | 96 |
| + Precipitation | 0.2109 | 2.41 | 102 | 0.258 | 7.12 | 96 |
| + Relative Humidity | 0.2006 | 1.38 | 102 | 0.2221 | 3.53 | 96 |
| + All drivers# | 0.3021 | 11.53 | 94 | 0.4331 | 24.63 | 68 |

† Basic Model: factors affecting $R_{t}$ include depletion of susceptibles, inter-epidemic factors. * Improved models include the basic model for $R_{t}$plus the respective drivers. # Improved model includes the drivers: mean temperature, diurnal temperature range, maximum Wind Speed, Precipitation and Relative Humidity (statistically significant and make sure to free from multicollinearity). ${\% \Delta R}^{2}$ measured the change in the explained variance (of total variance) from the model in comparison to the basic model. ${{\%}_{imp}\Delta R}^{2}$ measured the change in the explained variance (of variance explained by improved model) from the model in comparison to the basic model. i.e. $\% {\Delta R}^{2}=(R_{improved models}^{2}-R_{basic model}^{2})\times100$

**5. Supplementary figures**


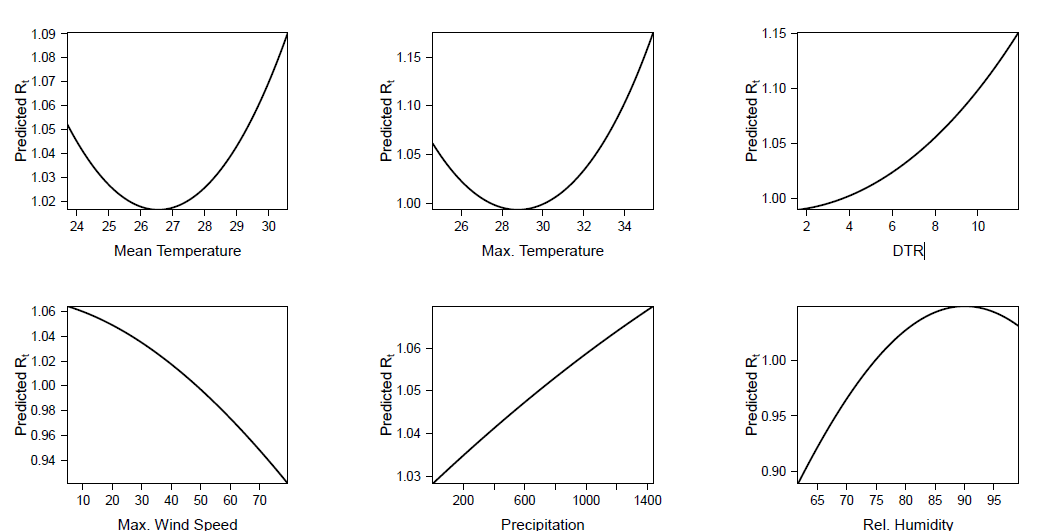


**Figure S1**: Estimated nonlinear relationship between the effective reproduction number $R_{t}$ and significant meteorological drivers in the univariate regression analysis (based on best-lag of the factors) for RSV transmission in Singapore.


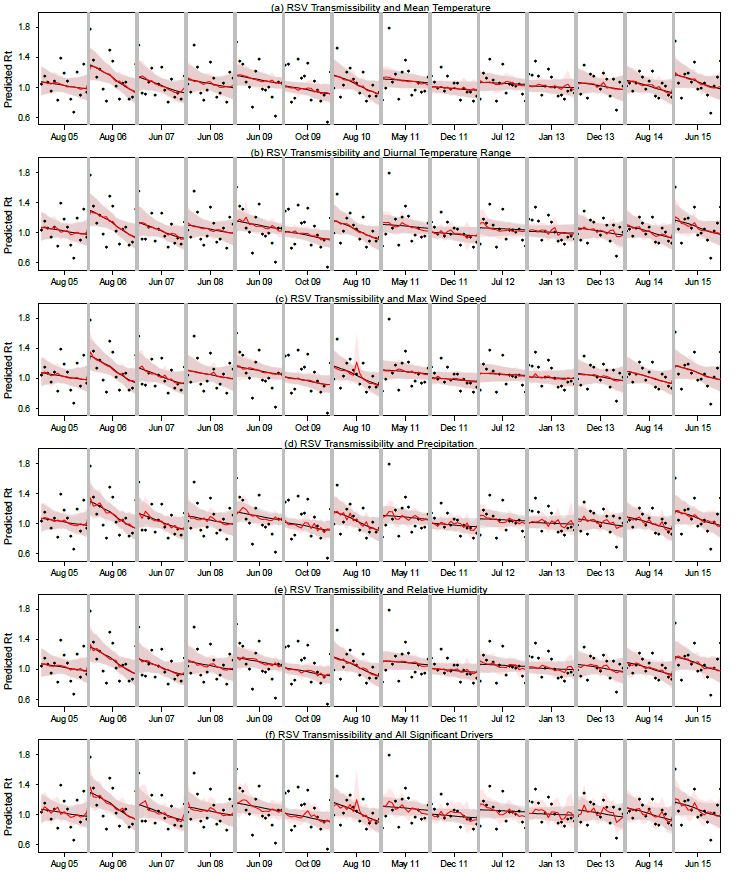


**Figure S2**: Effective reproduction numbers inferred from the RSV hospital admissions time series (black dots) and the predicted effective reproduction numbers from basic models with inclusion of depletion of susceptible and the inter-seasonal factors only (black lines) with 95% CI (light grey shaded area), and inclusion of meteorological drivers in addition to depletion of susceptible and the inter-seasonal factors (red lines) with 95% CI (light red shades). Total 11 epidemics (with 14 peaks) of RSV transmission in Singapore during 2005-2015. The difference between the black line and the red line illustrates the improvement in fitting due to inclusion of respective drivers. We used a maximum duration of 7 weeks to both side of peaks and a 5 days moving average window to smooth daily hospitalization data for Best lag model (Lag with highest R-square values) with lags of 0-14 days.

**References:**

21. Fraser C (2007) Estimating Individual and Household Reproduction Numbers in an Emerging Epidemic. PLoS ONE2(8): e758.
